# Improved diagnostic accuracy for ulnar-sided TFCC lesions with radial reformation of 3D sequences in wrist MR arthrography

**DOI:** 10.1007/s00330-021-08024-3

**Published:** 2021-05-18

**Authors:** Henner Huflage, Karsten Sebastian Luetkens, Andreas Steven Kunz, Nora Conrads, Rafael Gregor Jakubietz, Michael Georg Jakubietz, Lenhard Pennig, Lukas Goertz, Thorsten Alexander Bley, Rainer Schmitt, Jan-Peter Grunz

**Affiliations:** 1 Department of Diagnostic and Interventional Radiology, University Hospital Würzburg, Oberdürrbacher Straße 6, 97080 Würzburg, Germany; 2 Department of Trauma-, Hand-, Plastic- and Reconstructive Surgery, University Hospital Würzburg, Oberdürrbacher Straße 6, 97080 Würzburg, Germany; 3 Institute for Diagnostic and Interventional Radiology, Faculty of Medicine and University Hospital Cologne, University of Cologne, Kerpener Straße 62, 50937 Cologne, Germany; 4 Department of Radiology, University Hospital LMU Munich, Marchioninistrasse 15, 81377 Munich, Germany

**Keywords:** Wrist, Arthrography, Magnetic resonance imaging, Triangular fibrocartilage, Joint instability

## Abstract

**Objectives:**

Triangular fibrocartilage complex (TFCC) injuries frequently cause ulnar-sided wrist pain and can induce distal radioulnar joint instability. With its complex three-dimensional structure, diagnosis of TFCC lesions remains a challenging task even in MR arthrograms. The aim of this study was to assess the added diagnostic value of radial reformatting of isotropic 3D MRI datasets compared to standard planes after direct arthrography of the wrist.

**Methods:**

Ninety-three patients underwent wrist MRI after fluoroscopy-guided multi-compartment arthrography. Two radiologists collectively analyzed two datasets of each MR arthrogram for TFCC injuries, with one set containing standard reconstructions of a 3D thin-slice sequence in axial, coronal and sagittal orientation, while the other set comprised an additional radial plane view with the rotating center positioned at the ulnar styloid. Surgical reports (whenever available) or radiological reports combined with clinical follow-up served as a standard of reference. In addition, diagnostic confidence and assessability of the central disc and ulnar-sided insertions were subjectively evaluated.

**Results:**

Injuries of the articular disc, styloid and foveal ulnar attachment were present in 20 (23.7%), 10 (10.8%) and 9 (9.7%) patients. Additional radial planes increased diagnostic accuracy for lesions of the styloid (0.83 vs. 0.90; *p* = 0.016) and foveal (0.86 vs. 0.94; *p* = 0.039) insertion, whereas no improvement was identified for alterations of the central cartilage disc. Readers’ confidence (*p* < 0.001) and assessability of the ulnar-sided insertions (*p* < 0.001) were superior with ancillary radial reformatting.

**Conclusions:**

Access to the radial plane view of isotropic 3D sequences in MR arthrography improves diagnostic accuracy and confidence for ulnar-sided TFCC lesions.

**Key Points:**

*• In multi-compartment arthrography of the wrist, ancillary radial plane view aids assessability of the foveal and styloid ulnar-sided insertions of the triangular fibrocartilage complex.*

*• Assessment of peripheral TFCC injuries is more accurate with access to radial multiplanar reconstructions.*

*• Additional radial planes provide greater diagnostic confidence.*

## Introduction

**Fig. 1:**
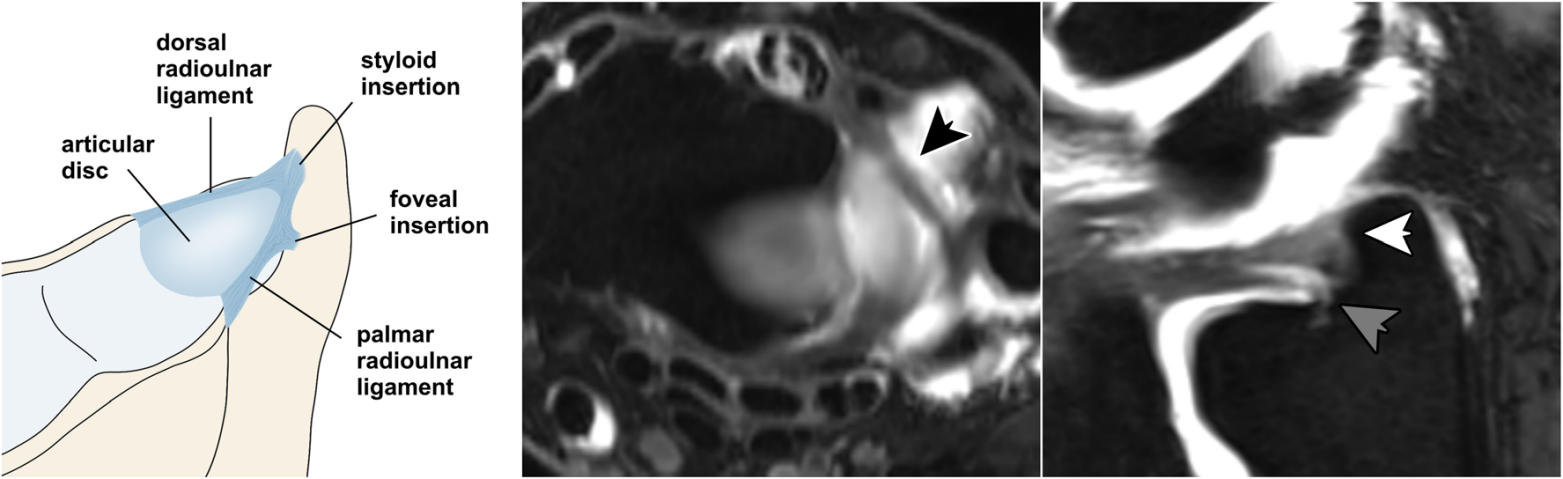
Schematic drawing of the triangular fibrocartilage complex demonstrates the delicate three-dimensional anatomy of the ulnar-sided periphery (left). Axial plane (middle) displays the course of the dorsal radioulnar ligament (black arrow), while radial reformatting of thin-slice 3D MRI (right) allows for visual differentiation of its foveal (grey arrow) and styloid insertion (white arrow)

With a two-layered, three-dimensional composition and multiple miniscule components contributing to its integrity, the triangular fibrocartilage complex (TFCC) is considered to be one of the most challenging regions from an imaging perspective. It consists of several conjoint parts with different vascularization patterns that influence the available treatment in case of discontinuity [1]. Originating from the sigmoid notch of the radius is the avascular articular disc, whereas the peripheral part is vascularized and consists of a deep and superficial lamina that inserts in the ulnar fovea and at the ulnar styloid process, respectively [2, 3]. The deep layer of the ulnar-sided TFCC is formed by the triangular ligament that arises from the convergence of the palmar and dorsal radioulnar ligaments. It functions as the main stabilizer of the distal radioulnar joint (DRUJ) during pronation and supination [4]. Contrastingly, the superficial layer is shaped like a hammock and assists the ulnocarpal ligaments in the transmission of axial forces between the distal forearm and proximal carpal row (Fig. 1). Fat tissue is usually discernible between the foveal and styloid lamina.

Pathologies of the TFCC commonly cause ulnar-sided wrist pain and can result from high-impact injuries, repetitive microtrauma, degeneration, systemic conditions (e.g. crystal deposition diseases) or combinations of the aforementioned [5, 6]. Besides clinical findings, hand surgeons rely heavily on imaging to indicate either conservative or operative treatment with MRI being the method of choice for three-dimensional wrist evaluation. To achieve the contrast-to-noise ratio necessary for a depiction of microstructural alterations of cartilage and ligaments, different MRI techniques can be applied: while plain T2-weighted sequences depend on effusion surrounding the rupture site and T1-weighted MRI after intravenous gadolinium application requires reactive hyperemia of the adjacent synovial tissue for lesion visualization, MR arthrography is capable of directly displaying TFCC discontinuity [7–9]. However, even in MR examinations after direct arthrography, radiologic standard planes frequently fail to depict the entirety of the TFCC due to its complex triangular shape and small component size. With the coronal plane being the most commonly used for TFCC assessment, peripheral lesions are especially difficult to visualize [10]. In a recent study, Götestrand et al [11] were able to show the general advantages of high-resolution 3D sequences over conventional 2D MRI for the visualization of the foveal TFCC attachment. Besides, isotropic 3D MRI can be used for multiplanar reformations with arbitrary orientation [12, 13]. Similar to angulated planes for the intrinsic carpal ligaments [14], radial reformatting has been suggested as a reasonable tool to assess TFCC lesions following CT arthrography in previous studies [15, 16].

Notwithstanding the establishment of thin-section 3D MRI and anatomically angulated reformatting for other body regions [17–19], radial multiplanar reconstructions (MPR) of isotropic 3D MRI sequences have not been evaluated for the ulnar side of the wrist. Therefore, this study aims to investigate the added diagnostic value provided by ancillary radial reformatting for TFCC assessment.

## Methods

### Study population

**Fig. 2:**
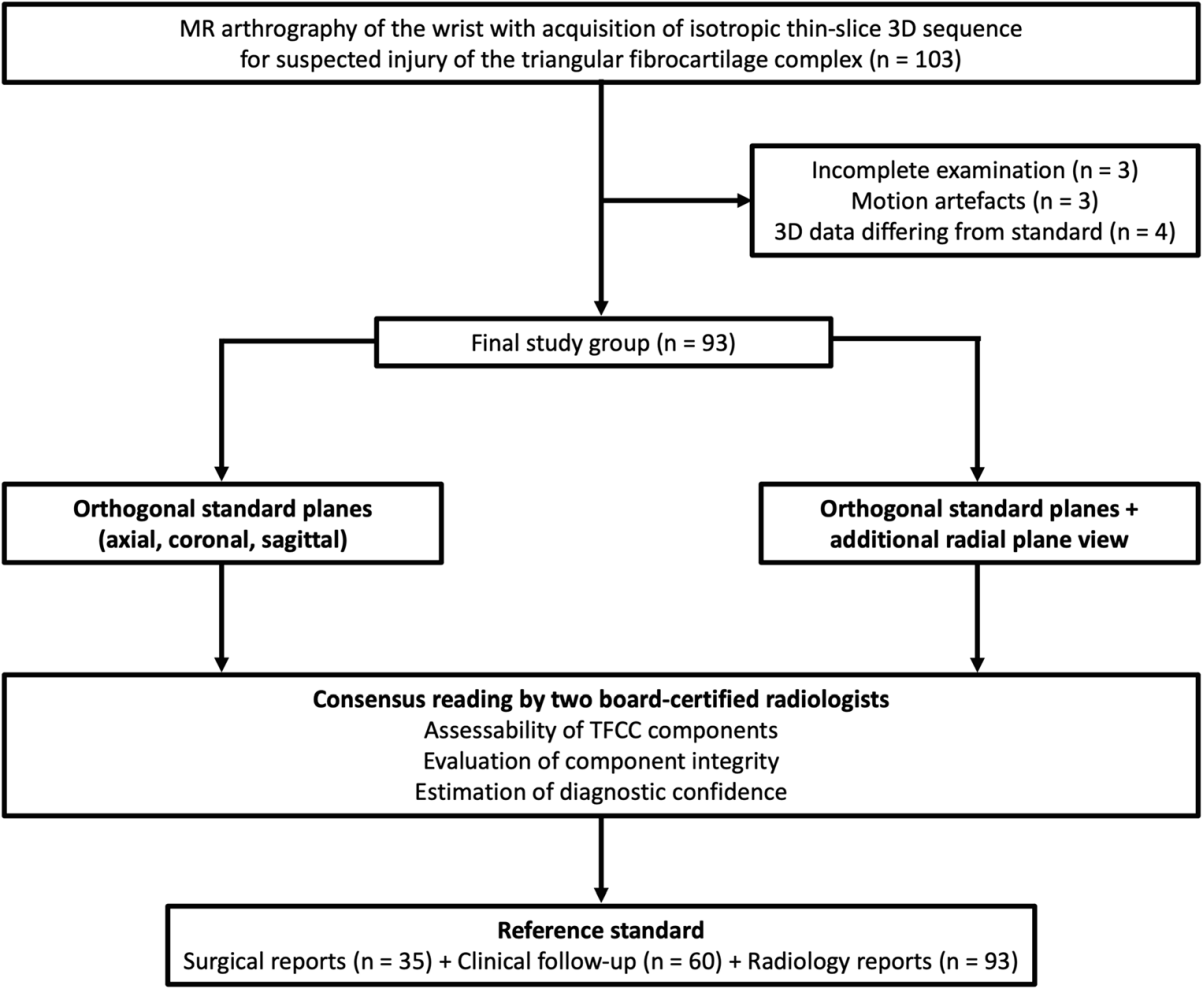
Flow chart for visualization of the study population, exclusion/inclusion criteria and method of data analysis

Informed consent was waived and permission for this retrospective study was obtained from the local ethics committee. MR arthrograms with acquisition of isotropic 3D sequences for suspected TFCC injury were available in 103 patients between August 2010 and October 2017. All patients received MR imaging immediately after undergoing fluoroscopy-guided multi-compartment arthrography of the wrist. Ten patients had to be excluded from the study for one of the following reasons: incomplete examination (*n* = 3), strong artefacts (*n* = 3), acquisition of 3D dataset differing from study standard (*n* = 4). As a result, the study group is comprised of 93 patients, including 44 women (47.3%), and had a mean age of 36.9 ± 13.8 years (range 17–73 years). In 49 cases (52.7%), the left wrist was scanned. Fifty-four patients reported a history of wrist trauma (58.1%). Summarizing exclusion and inclusion criteria, a flow chart depicts the patient population analyzed in this study (Fig. 2).

### Direct arthrography of the wrist

Wrist arthrograms were conducted under fluoroscopic guidance (Fluorospot Compact, Siemens Healthcare GmbH) by board-certified radiologists. Gadolinium diluted with sodium chloride (Magnograf 0.5 mmol/mL, Marotrast GmbH) was used for articular injection. As per department protocol, all procedures were performed with the patient in a prone position and the afflicted wrist extended above the head. In absence of communicating defects, the three main compartments of the wrist were injected with a contrast agent to detect isolated or combined injuries of the TFCC and the intrinsic carpal ligaments. Contrasting the midcarpal joint (approx. 2.0 mL), DRUJ (approx. 0.8 mL) and radiocarpal joint (approx. 2.5 mL) in succession was demonstrated to improve visualization of non-communicating lesions and increase overall diagnostic accuracy [20–22].

### MRI examinations

**Fig. 3:**
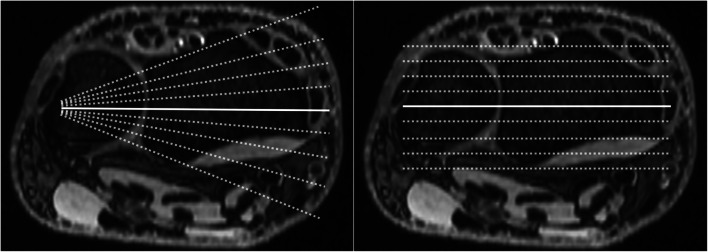
Multiplanar reformatting of isotropic thin-slice MRI in radial (left) and coronal (right) orientation based on axial planes through the distal radioulnar joint

Immediately after multi-compartment arthrography of the wrist, patients were transferred to the MRI suite for further imaging. Studies were performed on a 3.0 T scanner (Magnetom Prisma or Magnetom Skyra, Siemens Healthcare GmbH) with the patient adopting the so-called superman position also used for fluoroscopic scans. MRI examinations were conducted using a multi-channel phased-array coil dedicated to wrist imaging. Coronal acquisition of isotropic T2-weighted 3D Double Echo Steady State sequence (DESS; voxel size of 0.6 × 0.6 × 0.6 mm^3^) with axial and sagittal reformatting was mandatory for study inclusion and available in all 93 patients. Additional 2D sequences were acquired in accordance with the department standard but were not evaluated in the observer analysis. Radial plane view was prepared retrospectively and adapted to the triangular shape of the TFCC with the ulnar styloid as the center of rotation (Fig. 3). Section thickness for radial MPR was 2.0 mm with an angle of 5° between images.

### Image assessment

Two board-certified radiologists with seven years of musculoskeletal imaging experience collectively analyzed two datasets for each patient in randomized order using a commercially available picture archiving and communication system (Merlin, Phönix-PACS) installed on a conventional workstation with a certified diagnostic monitor. One set contained the coronally acquired 3D DESS sequence with axial and sagittal reconstructions, whereas radial reformatting was additionally available for assessment in the other dataset. Blinded to any patient information, observers were given three tasks for each read: first, to rate the assessability of the articular disc, the foveal and styloid attachment of the peripheral TFCC using a five-point scale (5 = excellent, 4 = good, 3 = moderate, 2 = fair, 1 = not diagnostic). Second, to evaluate the continuity of said parts in dichotomous fashion (0 = no lesion, 1 = lesion). Third, to state their overall diagnostic confidence (5 = total, 4 = high, 3 = moderate, 2 = slight, 1 = little to no confidence). Surgical reports were used as the reference standard whenever available (in 35 patients). Only in patients that did not undergo surgery, radiological reports by musculoskeletal imaging specialists (available in all 93 patients) in combination with clinical follow-up (in 60 patients) served as a standard of reference. Thereby, the latter describes the control examination that patients are usually advised to undergo 4–6 weeks after the initial assessment that included the MR arthrogram.

### Statistics

Dedicated statistical software (SPSS Statistics Version 27.0 for Mac, IBM) was used to perform data analyses. Normally distributed data is presented as mean ± standard deviation (SD); otherwise, we report frequencies and percentages with median values. Normal distribution was assessed with Kolmogorov-Smirnov tests. Paired categorical variables were compared using Wilcoxon signed-rank tests, while McNemar tests were performed for comparison of classification functions (e.g. specificity, sensitivity). Statistical significance was assumed for *p* values ≤ 0.05.

## Results

Subjective evaluation of the central articular disc provided excellent results in the far majority of patients irrespective of dataset (69.9% of standard examinations vs. 73.1% of examinations with radial plane view; *p* = 0.237). In contrast, assessability of the superficial (31.2% vs. 65.6%; *p* < 0.001) and deep (35.5% vs. 71.0%; *p* < 0.001) ulnar-sided layer of the TFCC was superior with addition of radial reformatting (Table 1).

**Table 1:** Collective evaluation of triangular fibrocartilage complex assessability by both radiologists (5 = excellent, 4 = good, 3 = moderate, 2 = fair, 1 = not diagnostic). Scale results are reported as absolute values (percentages)

Subjective assessability	Orthogonal standard planes			Additional radial plane view		
	Disc proper	Deep insertion	Superficial insertion	Disc proper	Deep insertion	Superficial insertion
5	65 (69.9%)	33 (35.5%)	29 (31.2%)	68 (73.1%)	66 (71.0%)	61 (65.6%)
4	24 (25.8%)	44 (47.3%)	42 (45.2%)	24 (25.8%)	23 (24.7%)	25 (26.9%)
3	2 (2.2%)	14 (15.1%)	20 (21.5%)	1 (1.1%)	4 (4.3%)	7 (7.5%)
2	1 (1.1%)	0	0	0	0	0
1	1 (1.1%)	2 (2.2%)	2 (2.2%)	0	0	0

**Fig. 4:**
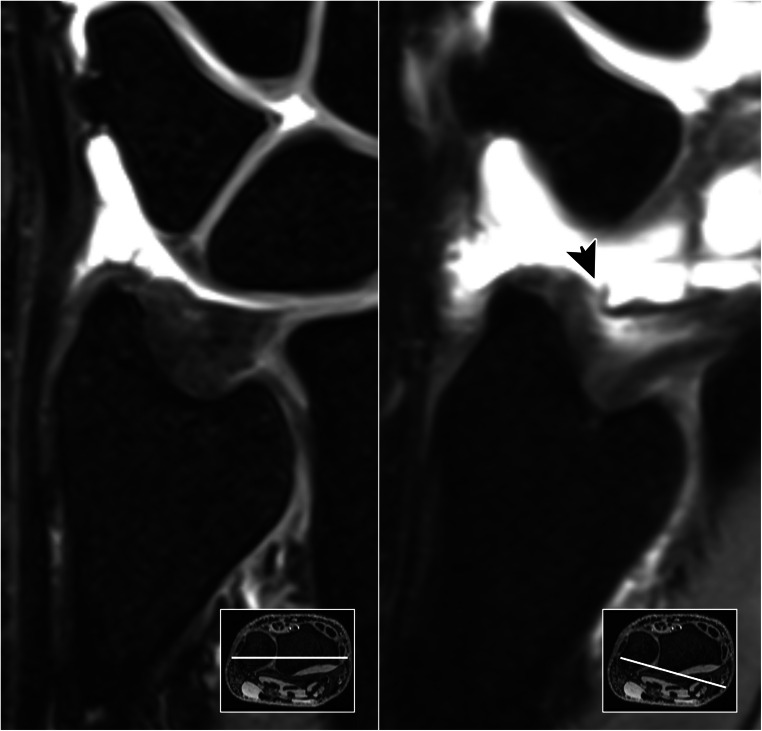
Without trauma, a 57-year-old woman reports to the surgery department for load-dependent ulnar-sided wrist pain. While standard coronal planes (left) were unable to visualize any form of TFCC injury, radial multiplanar reformatting (right) helped to identify a partial lesion of the superficial palmar radioulnar ligament (arrow). Not requiring refixation, the advised treatment was immobilization

**Fig. 5:**
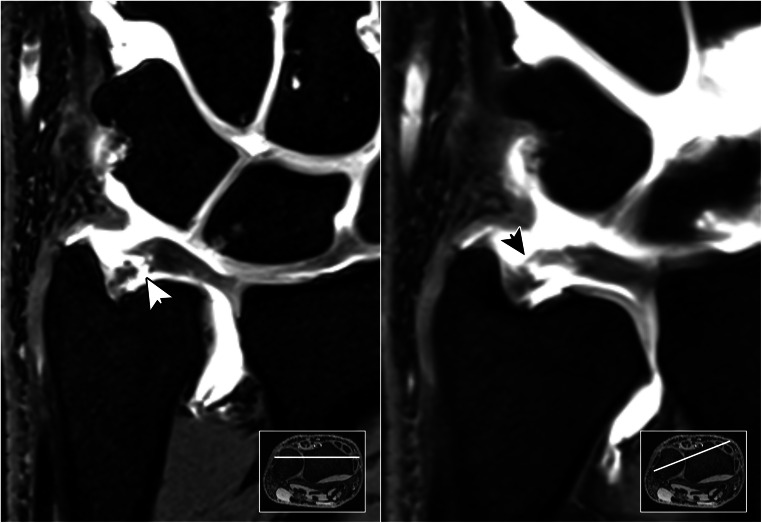
A 43-year-old man fell on his outstretched left hand. He noticed increasing pain on the ulnar side of the wrist. While no fracture was ascertained in radiography, orthogonal reformatting of MR arthrogram (left) suggested a complete tear in the ulnar portion of the TFCC (white arrow). Radial image reconstruction (right) revealed a high-grade partial tear with some dorsal fibers of the styloid lamina still intact (black arrow). Due to the confirmed discontinuity of the deep insertion, which functions as the main stabilizer of the distal radioulnar joint, refixation was recommended nonetheless

**Fig. 6:**
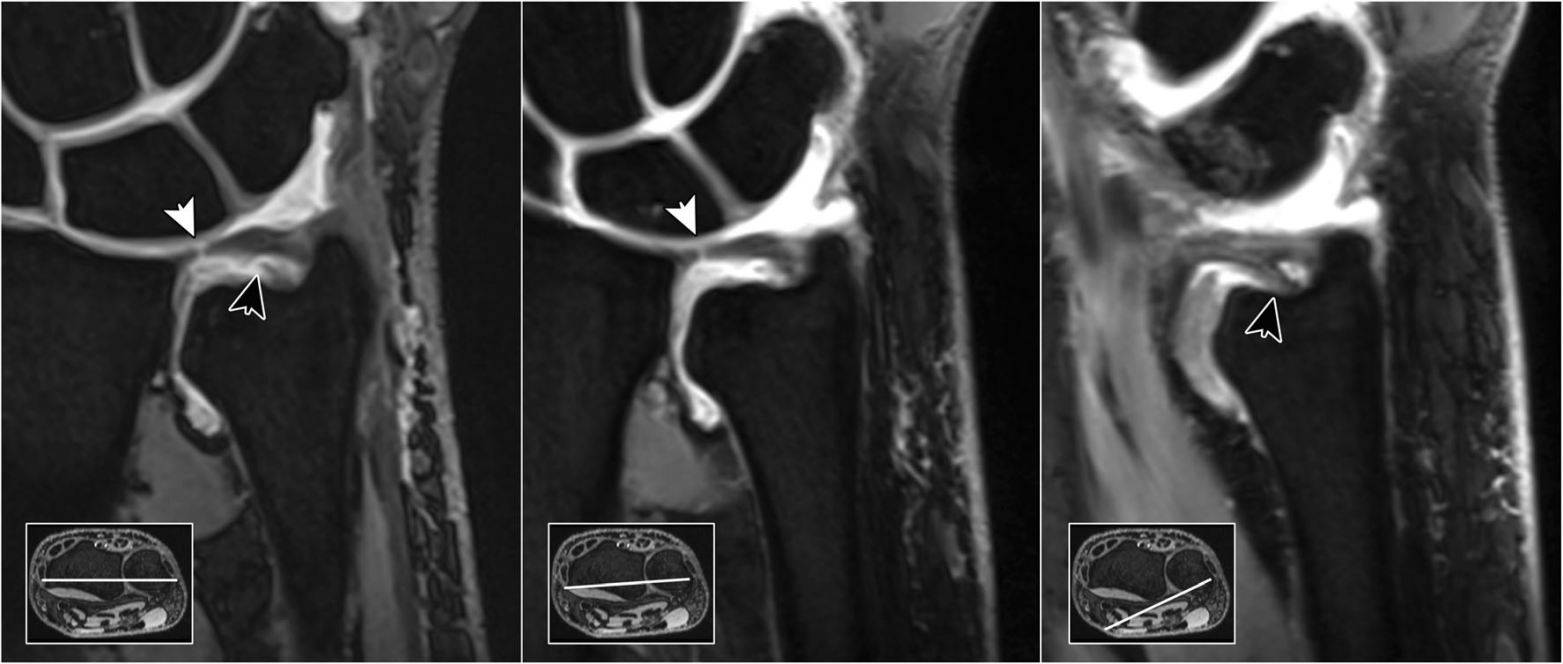
A 51-year-old man reports with ulnar-sided tenderness during pronation that has aggravated since a hyperextension trauma 5 weeks prior to the MR arthrogram. Standard coronal planes (left) reveal a pinhole defect in the central cartilage (white arrow). In addition, radiologists suspected a deep layer injury of the ulnar periphery (black arrow). Radial reformatting confirmed the central discus lesion (middle), while simultaneously ruling out the suspected discontinuity of the foveal insertion (right). Arthroscopy was performed for debridement and verified the integrity of the ulnar-sided TFCC attachments

In 93 MR arthrograms, 22 patients (23.7%) displayed lesions of the articular disc, while discontinuity of the styloid and foveal ulnar attachment was ascertained in 10 (10.8%) and 9 (9.7%) cases, respectively. With access to standard examination datasets, radiologists were able to correctly identify 20 lesions of the central disc, as well as 7 lesions each of the superficial and deep ulnar-sided layer, corresponding to sensitivities of 0.91, 0.70 and 0.78. With falsely assumed lesions in 4 (disc), 11 (styloid) and 13 (foveal) arthrograms, specificities of 0.94, 0.84 and 0.87 were achieved without radial planes. With radial reformatting available, sensitivity (0.91; 20/22 lesions detected) and specificity (0.93; 5 false positives) for articular disc injuries did not change (*p* > 0.99). For assessment of the ulnar-sided periphery, however, classification functions improved with the addition of radial MPR (Table 2): deep layer injuries were discovered more reliably (0.89 sensitivity; 0.94 specificity; 8/9 lesions detected; 5 false positives; *p* = 0.039), while superficial lesions of the peripheral TFCC were also identified with increased sensitivity and specificity (0.90 each; 9/10 lesions detected; 8 false positives; *p* = 0.016). Compared to datasets with standard examinations, overall diagnostic accuracy was superior for styloid (0.83 vs. 0.90; *p* = 0.016) and foveal attachment injuries (0.86 vs. 0.94; *p* = 0.039) when readers had additional access to the radial plane view (Figs. 4, 5, and 6).

**Table 2:** Indicators of diagnostic accuracy for lesions of the triangular fibrocartilage complex in standard planes and with ancillary radial multiplanar reconstructions

Classification functions	Orthogonal standard planes			Additional radial plane view		
	Disc proper	Deep insertion	Superficial insertion	Disc proper	Deep insertion	Superficial insertion
Specificity	0.94	0.87	0.84	0.93	0.94	0.90
Sensitivity	0.91	0.78	0.70	0.91	0.89	0.90
Accurarcy	0.94	0.86	0.83	0.92	0.94	0.90

Concerning diagnostic confidence, considerable improvement was recorded with radial MPR available (*p* < 0.001). Although high confidence was reported in the majority of arthrograms independent of dataset, total confidence votes (scale value 5) were more common with addition of radial planes (83.9% vs. 55.9%). Moderate or less confidence (scale value ≤ 3) was only declared if anatomically angulated reconstructions were not accessible (18.3%). Reader confidence is summarized in Table 3.

**Table 3:** Collective confidence assessment by both radiologists (5 = total, 4 = high, 3 = moderate, 2 = slight, 1 = little to no confidence). Scale results are reported as absolute values (percentages)

Diagnostic confidence	Orthogonal standard planes	Additional radial plane view
**5**	52 (55.9%)	78 (83.4%)
**4**	24 (25.8%)	15 (16.1%)
**3**	16 (17.2%)	0
**2**	1 (1.1%)	0
**1**	0	0

## Discussion

This study on 93 MR arthrograms of the wrist revealed that radial reformatting of isotropic 3D datasets with respect to the triangular shape of the ulnocarpal complex enhances diagnostic accuracy and confidence for lesions of the ulnar-sided TFCC attachments. Despite being a common cause of ulnar-sided wrist pain and impaired stability of the DRUJ, diagnosis of TFCC injuries remains a challenging task for radiologists. Multi-compartment arthrography with subsequent MRI is a well-established technique to analyze ligamentous and cartilaginous pathologies of the wrist⁠ [23, 24]. Advantages of directly injecting contrast agent into a joint compartment include articular distention and improved contrast-to-noise ratio, which aid the differentiation between the foveal and styloid insertion of the TFCC in partial-thickness tears⁠ [25]. Combinations of arthrography, thin-slice 3D sequences and anatomically angulated reconstructions are frequently implemented in knee⁠, shoulder⁠ and hip⁠ MRI [19, 26, 27]. For the TFCC, however, the benefits of preparing a radial plane view based on isotropic 3D MRI data acquisition have not been thoroughly evaluated.

Irrespective of the available reconstructions, diagnostic sensitivity, specificity and accuracy were at least good for all portions of the TFCC in this study, which is in line with previous literature [10, 28, 29]. Our results suggest that additional radial reformatting might be particularly useful for the detection and exclusion of lesions in the ulnar-sided periphery of the TFCC, whereas central disc defects were diagnosed with similar accuracy when radial MPR was not available. We assume that the partial volume effect resulting from the converging courses of the dorsal and palmar radioulnar ligaments towards the ulnar styloid process limits evaluation of continuity in the ulnar-sided periphery on standard orthogonal planes. For this reason, the disc proper may be assessable reliably, while the distal edges of the TFCC are not sufficiently represented on orthogonal reconstructions. Further substantiating this hypothesis, subjective ratings for assessability remained almost constant for the central cartilage but were considerably higher for the peripheral insertions when readers had access to the radial plane view. It has to be noted that wrist positioning can influence the assessability of the radioulnar ligaments. In pronation, particularly the laxity of the palmar radioulnar ligament may impair diagnostic evaluation [30]. However, while imaging in a neutral position may be beneficial for radioulnar ligament analysis, we believe based on clinical experience that wrist pronation is easier to maintain for the duration of the scan, resulting in less motion artefacts. Combining the image information of different wrist positions, dynamic evaluation in the form of cine MRI has the potential to simulate radial reformatting through actual radial-ulnar wrist deviation during scans. While clinical applicability of this technique has been shown in recent years [31, 32], cine MRI currently is only performed at few institutions.

Concordant with previous studies on CT wrist arthrography, diagnostic accuracy for central and peripheral lesions converged with additional radial reformatting [15, 16]. The deep peripheral layer of the TFCC includes the insertion of the triangular ligament into the ulnar fovea and functions as the main stabilizer between the ulnar head and sigmoid notch. Increased diagnostic accuracy when assessing this anatomic structure is of particular clinical relevance. Radiocarpal arthroscopy can only detect ruptures of the foveal ulnar insertion directly if the superficial layer is also disconnected from the ulnar styloid process because the trampoline and hook test may turn out negative otherwise. Considering that DRUJ arthroscopy remains a highly specialized procedure with limited applicability if the foveal attachment of the ulnar-sided TFCC is intact⁠, non-invasive assessment of stability is essential to decide the appropriate treatment option and prevent unnecessary surgical procedures [33]. With superior visibility of the TFCC periphery in radial MPR, an increase of overall diagnostic confidence was anticipated. The large amount of total confidence votes, however, might be beneficial for interdisciplinary exchange between radiologists and hand surgeons, as radiological reports may become more unambiguous with access to radial reformatting. Finally, despite providing a substantial advantage for the diagnosis of ulnar-sided TFCC injuries, the reconstruction of radial planes in postprocessing of isotropic 3D sequences requires little time and effort.

### Limitations

In accordance with the classifications of Atzei [34] (for ulnar-sided lesions) and Palmer [35] (for any type of TFCC injury), partial lesions of the disc proper, superficial and foveal insertions were not discerned from complete tears in this study. Using a dichotomous assessment approach for continuity, traumatic and degenerative alterations were not distinguished by observers. Lesions of the capsular attachments, meniscus homologue, ulnar collateral ligament, extensor carpi ulnaris tendon sheath and ulnocarpal ligaments were not in the scope of this study and therefore not evaluated. While two different 3.0 T scanners were used for wrist scans, potentially influencing the overall image quality, the acquisition parameters for the 3D DESS sequence were the same on both systems. Due to the high diagnostic accuracy of MR wrist arthrography that usually entails omission of additional arthroscopic evaluation if the radiological assessment turns out unequivocally negative for DRUJ instability, the number of surgical reports was limited in this study. For all 35 patients that underwent surgery, the respective report served as the sole standard of reference. Only if surgical correlation was not available, radiological reports by musculoskeletal imaging specialists and potential clinical follow-up were used to define an alternative reference standard [36]⁠. Compared to the literature⁠, however, surgical confirmation was rather common with 37.6% of patients undergoing arthroscopy in this study [7, 37, 38]. While the ulnocarpal complex was always evaluated diagnostically, not all surgical procedures were primarily performed for TFCC lesions, as unstable injuries of the intrinsic carpal ligaments were also treated in minimally invasive fashion.

## Conclusion

Adding radial plane view with the rotational center positioned at the ulnar styloid to the reconstruction algorithm for isotropic 3D sequences in MR arthrograms of the wrist improves diagnostic accuracy and confidence for peripheral TFCC lesions.

## Abbreviations

DESS: Double echo steady state
DRUJ: Distal radioulnar joint
MPR: Multiplanar reconstruction
SD: Standard deviation
TFCC: Triangular fibrocartilage complex

## Declarations

### Guarantor

The scientific guarantor of this publication is Henner Huflage (Huflage_H@ukw.de).

### Statistics and Biometry

No complex statistical operations were used in this manuscript.

### Methodology

• Retrospective

• Observational

• Performed at one institution
